# Biochemical and Proteomic Changes in the Roots of M4 Grapevine Rootstock in Response to Nitrate Availability

**DOI:** 10.3390/plants10040792

**Published:** 2021-04-17

**Authors:** Bhakti Prinsi, Chiara Muratore, Luca Espen

**Affiliations:** Department of Agricultural and Environmental Sciences—Production, Landscape, Agroenergy (DiSAA), Università degli Studi di Milano, I-20133 Milano, Italy; bhakti.prinsi@unimi.it (B.P.); chiara.muratore@unimi.it (C.M.)

**Keywords:** *Vitis*, mineral plant nutrition, perennial crop, nitrate, root growth

## Abstract

In agricultural soils, nitrate (NO_3_^−^) is the major nitrogen (N) nutrient for plants, but few studies have analyzed molecular and biochemical responses involved in its acquisition by grapevine roots. In viticulture, considering grafting, NO_3_^−^ acquisition is strictly dependent on rootstock. To improve the knowledge about N nutrition in grapevine, this study analyzed biochemical and proteomic changes induced by, NO_3_^−^ availability, in a hydroponic system, in the roots of M4, a recently selected grapevine rootstock. The evaluation of biochemical parameters, such as NO_3_^−^, sugar and amino acid contents in roots, and the abundance of nitrate reductase, allowed us to define the time course of the metabolic adaptations to NO_3_^−^ supply. On the basis of these results, the proteomic analysis was conducted by comparing the root profiles in N-starved plants and after 30 h of NO_3_^−^ resupply. The analysis quantified 461 proteins, 26% of which differed in abundance between conditions. Overall, this approach highlighted, together with an increased N assimilatory metabolism, a concomitant rise in the oxidative pentose phosphate pathway and glycolysis, needed to fulfill the redox power and carbon skeleton demands, respectively. Moreover, a wide modulation of protein and amino acid metabolisms and changes of proteins involved in root development were observed. Finally, some results open new questions about the importance of redox-related post-translational modifications and of NO_3_^−^ availability in modulating the dialog between root and rhizosphere.

## 1. Introduction

Among mineral nutrients, nitrogen (N) is the one required in the greatest amounts by plants, being an integral constituent of many organic compounds, such as nucleic acids, proteins, co-enzymes, chlorophyll, phytohormones and secondary metabolites [1]. In grapevine, N, after H, C and O, is usually the fourth most abundant element, representing 2–5% of plant biomass, and, in many cases, it is the most limiting factor for growth (i.e., vigor) and harvest yield [2]. Many studies conducted on grapevine highlighted the deep impact of N supply on plant reproductive capability as well as on grape berry metabolism and, finally, on berry composition [2,3,4,5,6]. In this view, the tight relationship between the rootstock/scion combination and N availability is emerging [7,8,9]. The N status of grapevine, like other perennial crops, depends on many factors, such as the acquisition of N from the soil, its distribution among annual organs and its reallocation from annual growth parts into both fruits and woody tissues [10,11,12,13,14]. In this context, the N content in grape berries, lost from the vineyard each year, has been estimated to be 2–3 kg of N per ton of fruit matter [2]. Nevertheless, a good management of N in vineyard must consider multifaceted aspects, in light of the fact that an excessive N supply can determine detrimental effects on the quality of grape berries and, therefore, of wine [4,5,8]. The achievement of this goal also comes via the improvement of the knowledge of the physiological, biochemical and molecular processes involved in the N metabolism of grapevine.

Although *Vitis* species could use different N forms, such as NO_3_^−^, NH_4_^+^, urea and amino acids, NO_3_^−^, usually the predominant N form in vineyard soils, is the primary source of N for grapevine [2,15,16]. Consistent with the fluctuating availability of this anion in soil solution (from 100 µM to 10 mM and more), plants have evolved different root uptake systems with different affinity (high-affinity transport systems, HATSs, and low-affinity transport systems, LATSs) and with both constitutive and inducible components [17,18,19]. The presence of HATSs rapidly responding to NO_3_^−^ availability has been recently demonstrated in grapevine [20,21]. According to the proton gradient requirement for sustaining the ≈2H^+^: 1NO_3_^−^ symporters, a concomitant induction of plasma membrane H^+^-ATPase in grapevine roots exposed to NO_3_^−^ has also been described [20]. Interestingly, the capability to induce NO_3_^−^ transporters in response to NO_3_^−^ availability is strictly dependent on the scion/rootstock combination [21]. 

In grapevine, N assimilation can occur in both roots and leaves [2,22]. The process is performed by the well-characterized pathway that involves the reduction of NO_3_^−^ to NH_4_^+^ through two steps catalyzed by nitrate reductase (NR) and nitrite reductase (NiR) and the subsequent assimilation catalyzed by glutamine synthetase (GS) and glutamate synthase (GOGAT), finally producing glutamic acid [1,22]. The contribution of the two organs depends on several factors, like N availability, C metabolism (i.e., availability of C skeletons), environmental conditions, tissue age and others [2,22,23,24,25]. Moreover, NO_3_^−^ assimilation shows several connections with other biochemical pathways and responses, among which are amino acid metabolism, redox status and pH homeostasis [2,26,27]. Recently, Cochetel and co-workers published the first paper describing transcriptomic changes occurring in the roots of Riparia Gloire de Montpellier and 1103 Paulsen rootstocks in relation to NO_3_^−^ availability [28]. Comparing these two genotypes, this study highlighted both specific responses and common changes in the gene expression patterns. Nitrogen-responsive genes involved in the uptake and assimilation of NO_3_^−^ as well as genes encoding enzymes of the oxidative pentose phosphate (OPP) pathway were identified as being influenced by NO_3_^−^ availability. According to the literature, the increase in OPP pathway activity is related to the typical metabolic adaptation of root tissues needed to satisfy the demand for reducing power [29,30]. In this context, the entity of the translocation of sugars from the photosynthetic tissues into roots affects the N assimilatory contribution of the two organs [2]. 

To our knowledge, to date, no proteomic investigation has studied the N responses in grapevine roots. Therefore, improvement of the information at translational and post-translational levels is desirable to better characterize the molecular and biochemical events evoked by changes in N availability in this perennial species. In the present work, we adopted biochemical and proteomic approaches to analyze, in the root of M4 grapevine rootstock, the responses occurring after the addition of NO_3_^−^ to the nutrient solution in hydroponics. This rootstock genotype was selected on the basis of its good response to abiotic stress [31,32,33,34,35], which is contributing to the expansion of its use in viticulture. The biochemical evaluations were devoted to verifying the role played by roots in the response to NO_3_^−^ resupply, monitoring key parameters. Moreover, since a holistic approach represents a good strategy to study the biological complexity [18,26,29], proteomic analysis was used to obtain a more comprehensive description of the first metabolic events involved in NO_3_^−^ acquisition in roots of *Vitis*.

## 2. Results and Discussion

The activation of NO_3_^−^ metabolism, which involves the modulation of specific transport systems and an assimilation pathway, is tightly linked to the external availability of the nutrient [20,21,29]. The rate at which plants induce these activities deeply affects their efficiency in using this mineral element in field conditions [36]. Moreover, studies performed on grapevine highlighted that, during the induction phase, the root organ plays an important role not only in N uptake from the soil, but also in N assimilation [2]. Starting from these considerations, the present work aimed at investigating in the roots of M4 grapevine rootstock the responses to the addition of 10 mM NO_3_^−^ in the hydroponic nutrient solution after a period of N starvation (see Materials and Methods for further details). First, key biochemical parameters were measured to define the timing of induction of the metabolic processes involved in the acquisition of NO_3_^−^. Afterwards, the changes in the root proteome were investigated.

### 2.1. Changes in Biochemical Parameters in Response to Nitrate

**Figure 1:**
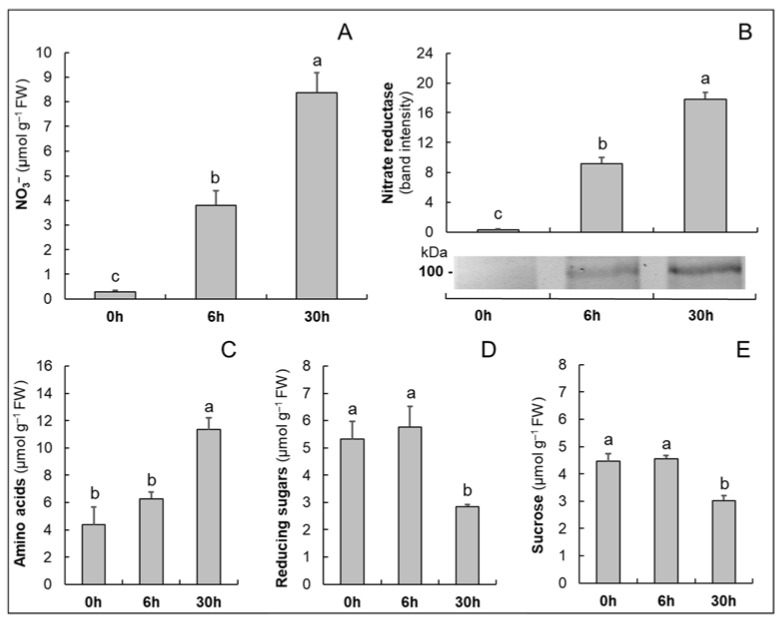
The time course of the changes in the contents of NO_3_^−^ (**A**), in the accumulation of nitrate reductase evaluated by immunoblot analysis (**B**), and in the contents of amino acids (**C**), reducing sugars (**D**) and sucrose (**E**) in the roots of M4 grapevine rootstock, previously grown in the absence of N for 8 d (0 h) and incubated for a further 6 and 30 h in the presence of 10 mM NO_3_^−^. The values are means ± SE (*n* = 3). Statistical significance was assessed by one-way ANOVA and the Holm–Šídák method. Different letters indicate significant differences (*p* ≤ 0.05).

Biochemical analyses of key parameters in the roots of M4 were performed in order to define plant adaptations to renewed NO_3_^−^ availability (Figure 1).

After the exposure of plants to NO_3_^−^, the contents of the anion in roots increased progressively within the first 30 h (Figure 1A), suggesting that NO_3_^−^ uptake and accumulation were activated early and maintained throughout the treatment period (30 h). According to the role of NO_3_^−^ as a signal for the induction of its assimilation [17], a parallel increase in NR abundance, evaluated by immunoblot analysis, occurred (Figure 1B). However, an increase in the content of amino acids was detected only after 30 h of treatment (Figure 1C), suggesting that a full activation of the NO_3_^−^ assimilatory pathway was reached at this time point. In support of this conclusion, a decrease in reducing sugar and sucrose contents (Figure 1D,E) was measured only after 30 h of treatment, probably resulting from an increase in the use of C skeletons to sustain N assimilation. 

This evaluation describes the typical metabolic changes involved in the acquisition and assimilation of NO_3_^−^ well [18,36]. Moreover, these results allowed us to define, in M4 grapevine rootstock, the timing of these responses, which, interestingly, is highly consistent with those described for other grapevine genotypes [20,21,28]. Starting with this information, we performed the proteomic analysis, comparing the roots of N-starved plants (control plants, 0 h) with those of plants grown in the presence of 10 mM NO_3_^−^ for 30 h.

### 2.2. Proteomic Analysis of M4 Root System

The proteomic analysis was performed by means of one-dimensional gel–liquid chromatography–mass spectrometry (GeLC–MS/MS), considering its suitability for the analysis of total proteomes in plant tissues [33,37]. Through this approach, it was possible to study the abundance of 461 proteins, with high reliability in identification and a good degree of comparability among samples and conditions (Table S1).

#### 2.2.1. Functional Distribution of the Identified Proteins

**Figure 2:**
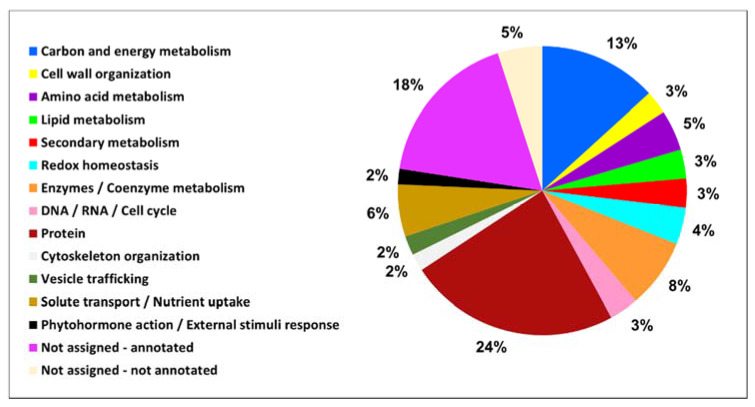
Functional distribution of all the identified proteins in the roots of M4 grapevine rootstock. Proteins were grouped in categories according to BIN ontology.

The functional classification of the identified proteins, reported in detail in Table S2, was conducted according to the MapMan4 ontology [38]. The identified proteins fell into 15 functional categories (Figure 2). Many of them belonged to four categories, “Protein” (24%), “Not assigned-annotated” (18%), “Carbon and energy metabolism” (13%) and “Enzymes/Coenzyme metabolism” (8%). Regarding the proteins that fell into the “Not assigned-annotated” category, in order to better define their functional role, a supplemental investigation was performed, using the UniProt database (https://www.uniprot.org/, accessed on 1 December 2020) and the basic local alignment search tool (BLAST). However, no information, or very fragmentary information, was available for proteins classified in the “Not assigned-not annotated” group, which corresponded to 5% of total identified proteins.

**Figure 3:**
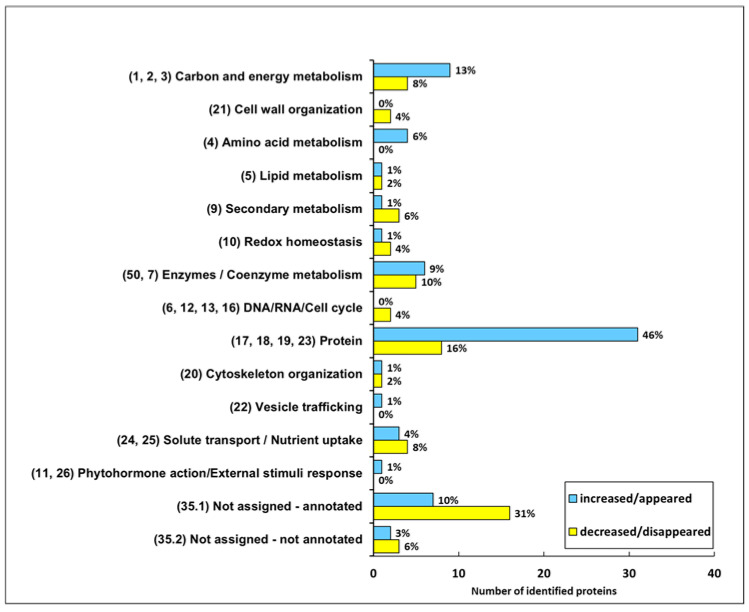
Functional distribution of the differentially accumulated proteins in roots of M4 grapevine rootstock after exposure of the plants to 10 mM NO_3_^−^. Proteins were grouped in categories according to BIN ontology (codes reported in brackets). The percentage refers to the total number of proteins having the same trend: blue bars, proteins that increased/appeared; yellow bars, proteins that decreased/disappeared.

Statistical analysis highlighted 119 proteins that significantly changed in abundance after the exposure of the plants to 10 mM NO_3_^−^ for 30 h. Figure 3 shows their functional distribution. Among these proteins, 68 increased or appeared while 51 decreased or disappeared. Almost a half (46%) of the proteins that increased in abundance belong to the “Protein” category. Interestingly, some other functional categories included proteins that were accumulated in a higher abundance in response to NO_3_^−^ supply, such as “Carbon and energy metabolism”, “Not assigned-annotated”, “Enzyme/Coenzyme metabolism” and “Amino acid metabolism” (13%, 10%, 9% and 6%, respectively). However, many of the proteins that decreased or disappeared were classified into the categories “Not assigned-annotated”, “Protein”, “Enzyme/Coenzyme metabolism” and “Carbon and energy metabolism” (31%, 16%, 10% and 8%, respectively).

Overall, the functional distribution of the NO_3_^−^-responsive proteins is consistent with the typical adaptations described in plants, and specifically in root tissues, in response to the availability of this nutrient [26,39,40]. Among them, modifications are evident in pathways related to the production of energy and C skeletons as well as broad changes in protein metabolism. In addition, several other metabolic traits were affected by the nutritional treatment. In details, Table 1 includes all the proteins that changed in abundance in response to NO_3_^−^.

#### 2.2.2. Proteomic Changes Involved in Nitrogen Acquisition and in Carbon and Energy Metabolism

The proteomic analysis revealed the appearance of ferredoxin-nitrite reductase (#246) in the roots of plants exposed to NO_3_^−^. According to the increases in NR abundance and in amino acid levels (Figure 1B,D), this result confirms the activation of the primary N assimilation pathway. Moreover, the appearance of ferredoxin-NADP reductase (#136) and glucose-6-phosphate 1-dehydrogenase (#323), as well as the dramatic increase in 6-phosphogluconate dehydrogenase (#64), is in agreement with a higher metabolic demand for NADPH, that in non-photosynthetic tissues is fulfilled by the OPP pathway [26,29]. However, cytosolic glutamine synthetase (#29) decreased in abundance in response to NO_3_^−^, suggesting a reduction in N recycling. This behavior probably mirrors the effect of NO_3_^−^ availability on the plant N status, and the consequent change in protein and amino acid catabolism [41].

At the same time, several glycolytic enzymes, such as fructose-bisphosphate aldolase (#28), phosphoglycerate kinase (#23), phosphoglycerate mutase (PGM, #154) and enolase (#81), increased in abundance in response to NO_3_^−^, according to a higher demand for C skeletons needed for N assimilation [42]. Among them, the peculiar role played by PGM that, even if it catalyzes a non-rate-limiting step in glycolysis, is involved in the recycling of glyceraldehyde-3P produced by the OPP is highlighted [26,29].

Interestingly, the concomitant decrease in the E1 component of pyruvate dehydrogenase (#122) suggests that an activation of anaplerotic reaction(s) occurred, rather than an upsurge of carbon oxidation through the Krebs cycle. In this view, it could be observed that the levels of the β subunit of ATP synthase (#2), mitochondrial aldehyde dehydrogenase (#11), NADH-cytochrome b5 reductase (#119) and NAD(P)H dehydrogenase (#110) also decreased after the addition of NO_3_^−^. Once again, this behavior could be ascribed to a shift in the balance between N recycling and assimilation.

In support of the above results, the activation of C metabolism in roots was accompanied by a decrease in the content of reducing sugars and sucrose (Figure 1D,E). In this view, it is interesting to observe that only in roots of plants exposed to NO_3_^−^ was a sucrose synthase (#14) detectable, an enzyme that plays a pivotal role in determining the sink strength necessary to import photoassimilates from the source organs [43]. This result suggests that the sink strength of the root system increases during the early phases of exposure to NO_3_^−^. Hence, it is possible that this response could have a role in the scion/rootstock relations and in the N use efficiency of different grafting combinations.

#### 2.2.3. Proteomic Changes Involved in Protein and Amino Acid Metabolism

The present study found that many proteins involved in protein synthesis, modification and degradation changed in abundance in response to NO_3_^−^ availability (Table 1). These include ribosomal proteins (#300, #126, #217, #443, #375) as well as initiation (#37) and elongation factors (#407, #149, #128). These changes agree with recent literature that highlights a resumption of these metabolic activities among the responses of N-starved plants to renewed availability of NO_3_^−^, in Arabidopsis (*Arabidopsis thaliana* L.) and maize (*Zea mays* L.) [26,39,40]. According to an activation of protein synthesis, a few heat shock proteins (#452, #1, #370, #317, #107), known to be involved in protein folding [44], were also more abundant in NO_3_^−^-treated plants. At the same time, the proteomic analysis revealed a different accumulation of proteins operating in the proteasome (#147, #420, #388) and in ubiquitination (#145, #379), suggesting an increase in protein turnover. 

Conversely, several proteases, such as two carboxypeptidases (#93, #195), a putative cysteine protease RD21B (#151) and an aspartic proteinase A1 (#26), decreased in abundance after the reintroduction of NO_3_^−^. Considering that some of these proteins have extracellular localization, these results support the hypothesis that during N starvation, roots are able to intensify the exudation of proteases, probably in order to mobilize N from peptides and proteins in the rhizosphere [45]. Interestingly, the secretion of such enzymes has also been observed in the absence of microorganisms [45], and, therefore, it is possible that it occurs in plants grown in hydroponic systems.

The metabolism of a few amino acids seemed to be positively affected upon NO_3_^−^ resupply. The proteomic analysis revealed an increase in the abundance of enzymes involved in the biosynthesis of methionine (#3, 5-methyltetrahydropteroyltriglutamate-homocysteine S-methyltransferase; #38, adenosylhomocysteinase), isoleucine (#73, acetohydroxy-acid reductoisomerase) and serine (#30, D-3-phosphoglycerate dehydrogenase). Moreover, an increase in the level of phospho-2-dehydro-3-deoxyheptonate aldolase (#140) was detected, being the first enzyme of the shikimate pathway, which is involved in the synthesis of aromatic amino acids. This result is in agreement with studies on other plant species that describe an activation of the synthesis of amino acids in order to sustain the reactivation of protein synthesis [26,40,46]. 

#### 2.2.4. Other Biochemical Functions Affected by Nitrate Resupply

A few enzymes involved in cell wall lignification, such as a caffeoyl-CoA O-methyltransferase (CCOMT, #131) and an omega-hydroxy-palmitate O-feruloyl transferase (OHFT, #54), decreased in abundance in response to NO_3_^−^ availability. It is interesting to observe that the late embryogenesis abundant protein Lea14-A (#215), described as able to positively regulate the deposition of lignin under stress conditions [47], followed a similar trend, as well as a glucan endo-1,3-beta-D-glucosidase (#105). These results are consistent with the well-documented reprogramming of the root development process that occurs in response to NO_3_^−^ [39,48,49]. Moreover, the different behaviors of two peroxidases (POXs, #208, #345) are noteworthy, which showed an opposite response to NO_3_^−^. Although a role of POX in the lignification process was proposed, the trends of CCOMT and OHFT strongly weaken this hypothesis and suggest a context in which this process seems reduced. However, considering the very multifaceted role of these enzymes, it is interesting to highlight that an increase in peroxidase and polyphenol oxidase (PPO) activities was described in grapevine during the adventitious rooting process [50]. Our proteomic analysis identified a PPO (#314) positively affected by NO_3_^−^ exposure, highlighting a possible analogy.

Other proteins involved in phenolic metabolism changed in abundance in response to NO_3_^−^. Among them, a decrease in chalcone isomerase (#106), an enzyme that catalyzes the isomerization of naringenin chalcone to naringenin flavanone in the flavonoid pathway, was observed. This result agrees with the well-known reduction in phenylpropanoid metabolism occurring after NO_3_^−^ resupply to N-starved plants [26,51,52,53,54]. At the same time, we observed an increase in trans-resveratrol di-O-methyltransferase (#41), which suggests different effects on specific traits of the phenylpropanoid metabolism. This enzyme catalyzes the formation of pterostilbene from resveratrol, a compound with antimicrobial and antifungal properties [55,56]. The specific role of this molecule in roots remains to be clarified, however, it was proposed that its increase upon high N availability could participate in promoting root relations with the rhizosphere community. Although further studies are needed, attention could be focused on the interplay occurring between plants and other organisms in the soil. In this view, it is interesting to note that our proteomic analysis identified a proline iminopeptidase (#265) that decreased in abundance in response to NO_3_^−^. Considering that this peptidase is induced by bacteria and seems to participate in virulence [57], our results suggest that NO_3_^−^ availability could reduce root susceptibility to pathogens.

Moreover, our proteomic analysis revealed changes of a few typical stress-responsive proteins. Some of them, such as major allergen Pru ar 1 (#199), chitin-binding type-1 (#395) and PHB (#42, #91), decreased, whilst others, such as MLP-like protein 34 and 43 (#89, #165) and annexin D2 (#46), increased in response to NO_3_^−^. Given the paucity of the current information for many of them [58], it is difficult to fully understand the biological meaning of these results. Recently, Wang and co-workers [59] provided new information about MLP-like protein 43, highlighting its participation in the drought responses mediated by ABA. In detail, these authors described the involvement of this protein in the response to oxidative stress as well as in the modulation of primary metabolism. Further analyses may help to clarify possible roles of this protein in the metabolic changes induced by NO_3_^−^. Although, for annexin, an interesting role in the differentiation/growth processes at the membrane level is emerging [60], and in view of the morphological responses evoked by NO_3_^−^ in root system, further work is necessary to define its specific role in grapevine rootstock.

The available literature describes changes in the expression of genes involved in redox metabolism upon NO_3_^−^ provision in Arabidopsis [26,39]. Our study revealed that NO_3_^−^ positively affected a glutaredoxin-dependent peroxiredoxin (#269) and cystathionine beta-synthase family protein a (#76), whilst another glutaredoxin-dependent peroxiredoxin (#185) and a thioredoxin h-type (#330) decreased. Considering their involvement in the regulation of the oxidative state of sulfhydryl groups and in the S-glutathionylation of proteins [61,62], this result underlines the crucial role of the redox system in the modulation of the metabolic adjustment evoked by increased NO_3_^−^ availability. Reinforcing this hypothesis, we also found four glutathione transferases (#108, #337, #448, #232) that showed an upsurge in abundance in response to NO_3_^−^ supply. Interestingly, future redox proteomics studies could be very useful to better clarify the role of redox-related post-translational modifications in response to N supply in plants.

Overall, our results highlight the important role of roots in the plant adaptations to changes in N availability. As previously described in other plant species, this study confirms that grapevine roots show a typical metabolic activation involved in the acquisition of this macronutrient. Moreover, this investigation brings evidence of interesting relationships between N availability and root–rhizosphere dialog. This new knowledge paves the way for further studies aimed at characterizing key factors involved in N use efficiency in this pluriannual species. Other interesting aspects that deserve attention in the future are the plant responses in field conditions, the adaptability to N availability in different graft combinations and the final outcomes in grape quality.

## 3. Materials and Methods

### 3.1. Plant Material and Nutritional Treatments

**Figure 4:**
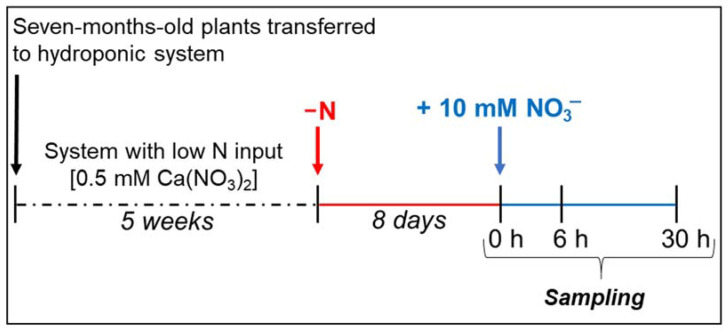
Growth experiment design and nutritional treatments. Further details are reported in the Material and Methods.

The M4 [(*V. vinifera* × *V. berlandieri*) × *V. berlandieri* cv. Resseguier no. 1] grapevine rootstock genotype was obtained from Vitro Hellas (Niseli Alexandreia, Greece, https://www.vitrohellas.gr/en/home, accessed on 1 August 2020). A scheme of the experimental design is shown in Figure 4. Plants, previously grown in peat soil, were flared, gently washed to remove residual particles of soil from the roots and then transferred to a hydroponic system. The experiments were conducted in a growth chamber with a 16/8 h day/night regime, at 26/22 °C, constant relative humidity of 65% and PPFD of 300 µmol m^−2^ s^−1^. All hydroponic solutions were continuously aerated by an electric pump. To allow adaptation to hydroponic conditions, plants were grown for 5 weeks in a nutrient solution (0.77 mM K_2_SO_4_, 0.65 mM MgSO_4_, 0.51 mM KH_2_PO_4_, 0.4 mM CaSO_4_, 100 µM Fe-EDTA, 50 µM KCl, 10 µM H_3_BO_3_, 1 µM MnSO_4_, 0.5 µM CuSO_4_, 0.5 µM ZnSO_4_, 0.35 µM Na_2_MoO_4_, pH = 6.1) containing 0.25 mM Ca(NO_3_)_2_ (i.e., low N input). The solution was replaced weekly. After this period, the plants were transferred into new nutrient solutions without N. After 8 days of N starvation (control plants, 0 h), plants were transferred to a fresh growing solution containing 10 mM KNO_3_. The start of the experiments coincided with the start of the light period. The plants were sampled at 0 h and after 6 and 30 h of treatment. Roots were rinsed with water, blotted with paper towels, and then immediately frozen in liquid N_2_. Each biological sample was composed of roots collected from three plants. Samples were stored at −80 °C.

### 3.2. Determination of the Contents of Nitrate, Amino Acids, Sucrose and Reducing Sugars

Nitrate was extracted from the tissues by homogenizing the samples in 4 volumes of distilled water and heating at 50 °C for 15 min. The homogenate was centrifuged at 12,000× *g* for 20 min to obtain a clarified supernatant. An aliquot of the supernatant was used for the determination of NO_3_^−^ concentration, according to Cataldo et al. [63].

Amino acids and total sugars were extracted in perchloric acid (PCA) as previously described by Meggio and co-workers [31]. The contents of total amino acids were measured by the ninhydrin method [64]. The contents of total soluble sugars were determined by boiling an aliquot of the PCA extract for 1 h before neutralization. Sugar concentrations were then measured according to the colorimetric method of Nelson [65]. All the analyses were replicated on three independent biological samples (*n* = 3) and compared by the ANOVA test (*p* < 0.05, Holm–Šídák method).

### 3.3. Protein Extraction

Protein extraction was performed as previously described [33]. Briefly, frozen powdered samples (1 g) of three biological samples for each experimental condition (*n* = 3) were finely powdered in liquid N_2_ using a pestle and mortar, adding 5% (*w/w*) of polyvinylpolypyrrolidone. The total protein fraction was extracted by adding 5 volumes of phenol and an adequate volume of aqueous buffer [0.7 M sucrose, 10 mM Na_2_-EDTA, 4 mM ascorbic acid, 0.2% (*v/v*) Triton X-100, 1 mM PMSF, 0.1 mg mL^−1^ Pefabloc and 0.4% (*v/v*) β-mercaptoethanol]. The protein fraction was then purified by methanol-based and acetone precipitation [66]. The final pellet was then dissolved in SDS buffer [150 mM Tris-HCl pH 6.8, 10% (*w/w*) glycerol, 2% (*w/w*) sodium dodecyl sulfate (SDS), 2% (*v/v*) β-mercaptoethanol] and incubated at 95 °C for 5 min. The sample was centrifuged at 10,000× *g* for 10 min and the supernatant stored at −80 °C until further use. The protein concentration was determined by the 2-D Quant Kit (GE Healthcare Europe GmbH, Freiburg, Germany).

### 3.4. Immunoblot Analyses

Protein samples (10 µg) were diluted with a volume of SDS buffer with 0.01% (*w/v*) bromophenol blue, heated for 5 min at 95 °C and then separated by SDS-polyacrylamide gel electrophoresis (SDS-PAGE) using 10% (*w/v*) polyacrylamide gel [67]. The samples were then electrophoretically transferred onto a polyvinylidene difluoride (PVDF) filter using a semidry blotting system (NovaBlot, Pharmacia, Sweden) with a buffer containing 10 mM 3-cyclohexylamino-1-propanesulphonic acid (CAPS, pH 11 with NaOH) and 10% (*v/v*) methanol. Filters were blocked for 1 h with TBS-T buffer [50 mM Tris–HCl (pH 7.6), 200 mM NaCl, and 0.1% (*v/v*) Tween 20] supplemented with 3% (*w/v*) albumin. The TBS-T buffer was used as an incubation medium throughout the procedure. Filters were incubated overnight at 4 °C with primary polyclonal antibodies against nitrate reductase using a 1:1,250 dilution (Agrisera, Vännäs, Sweden, AS08 310). After washing with TBS-T, the filters were incubated for 2 h at room temperature with a secondary antibody (alkaline phosphatase-conjugated anti-rabbit immunoglobulin G). The blot was developed with nitroblue tetrazolium and 5-bromo-4-chloro-3-indolyl phosphate (FAST BCIP/NBT, Sigma). The analysis was performed on three biological samples (*n* = 3), and the quantification of the signals was conducted through densitometric analysis by using the software ImageJ (https://imagej.net/, accessed on 1 August 2020).

### 3.5. Gel Electrophoresis and In-gel Digestion

Gel electrophoresis, in-gel digestion and mass spectrometry analyses were performed as previously described [40], with the following refinements. Briefly, 30 µg of proteins were separated on an SDS-PAGE on 16% (*w/v*) polyacrylamide gel [67], until samples ran a 3 cm length. After Coomassie brilliant blue staining, the blank portions of the gels as well as the regions above 250 kDa or below 12 kDa were removed. Each line was cut into 3 regular slices (10 × 10 × 0.75 mm). Each slice was then treated as an independent sample. In-gel digestion was performed according to Prinsi and co-workers [40]. The extracted peptides were finally dissolved in 0.1% (*v/v*) formic acid (FA).

### 3.6. Mass Spectrometry Analysis

All mass spectrometry experiments were conducted with an Agilent 6520 Q-TOF mass spectrometer equipped with an HPLC Chip Cube (Agilent Technologies, Cernusco sul Naviglio, Italy), as previously described [33], with some refinements. In detail, the peptides were eluted by applying a 100 min non-linear gradient of acetonitrile from 5% to 50% (*v/v*) in acidic conditions (FA 0.1% *v/v*) at 0.4 µL min^−1^. The mass spectrometer was run in positive ion mode and MS scans were acquired over a range from 300 to 3000 mass-to-charge ratio (m/z) at 4 spectra s^−^^1^. MS/MS scans were acquired over a range from 50 to 3000 mass-to-charge ratio (m/z) at 3 spectra s^−^^1^. Precursor ions were selected by auto-MS/MS with a maximum of 4 precursors per cycle and active exclusion set at 2 spectra for 0.1 min. Sample profiles were reconstructed by combining the chromatograms obtained for all three slices into which they were divided. Analysis of MS/MS spectra was performed by Spectrum Mill MS Proteomics Workbench (Rev B.04.00.127, Agilent Technologies). Carbamidomethylation of cysteine was set as a fixed modification while the oxidation of methionine was a variable modification. Trypsin was selected as the enzyme for digestion, accepting 2 missed cleavages per peptide. For spectrum interpretation, the search was conducted against the *Vitis* (ID 3603) protein database (December 2020, 167,581 entries) downloaded from UniProtKB/Swiss-Prot (http://www.uniprot.org/, accessed on 1 February 2021) and concatenated with the reverse one. The mass spectrometry proteomics data have been deposited in the ProteomeXchange Consortium [68] via the PRIDE partner repository with the data set identifier PXD025212. The threshold used for protein identification was peptide false discovery rate (FDR) ≤ 1% and number of unique peptides per protein ≥ 2. Peptide quantification was obtained as the spectrum intensity (SI) of the precursor. Protein quantification was obtained by summing the SIs of all the identified peptides in the protein. Protein abundance was normalized as the percentage with respect to the abundance of all validated proteins in the sample (%(SI)), summing all validated peptides in the 3 slices [33]. The analysis was performed using three biological samples for each condition (*n* = 3). Statistical significance was assessed by a Student’s *t*-test (*p* < 0.05). The identified proteins were classified into metabolic functional categories according to the MapMan4 BIN ontology [38].

## Figures and Tables

**Table 1:** Proteins differentially accumulated in response to 10 mM NO_3_^−^ for 30 h in roots of M4. Proteins are grouped according to functional classification (Figure 3). Subtitles report functional categories and their bin codes. **#**: identification number (group). ***UIP***: number of unique identified peptides. ***Score***: MS/MS search score. **Δ(*nit/con)***: fold changes expressed as the ratio between the protein abundance in NO_3_^−^-treated plants (nit, 30 h) and in the control N-starved plants (con, 0 h). **Blue cells**: proteins that increased in abundance. **Yellow cells**: proteins that decreased in abundance. **s**: statistical significance assessed by Student’s *t*-test (*n* = 3) (* *p* < 0.05, ** *p* < 0.01). **^a^**: protein annotated by BLAST. In *italic*s: additional information from UniProt. **New**: not detected in control plants; **d.**: disappeared, not detected in NO_3_^−^-treated plants.

#	Accession Number	*UIP*	*Score*	Protein Name	Δ *(nit/con)*	s
**Carbon and energy metabolism (1, 2, 3)**						
28	**A5B118**	12	167.9	Fructose-bisphosphate aldolase	**2.01**	*
23	**A5CAF6**	12	191.3	Phosphoglycerate kinase	**1.83**	*
122	**F6I1P0**	6	80.5	Pyruvate dehydrogenase E1 component subunit beta	**0.22**	**
251	**A5AY34**	4	45.9	Oxidored_q6 domain-containing protein	**4.34**	*
2	**A5AYU8**	18	330.6	ATP synthase subunit beta	**0.63**	**
150	**D7T300**	6	54.9	ATP synthase subunit O, mitochondrial I ^a^	**new**	*
14	**F6HGZ9**	14	160.9	Sucrose synthase	**new**	*
257	**Q1PSI9**	4	43.7	L-idonate 5-dehydrogenase	**15.59**	**
323	**A0A438D9B1**	3	36.8	Glucose-6-phosphate 1-dehydrogenase	**new**	**
169	**A0A438HWY8**	5	66.2	Probable 6-phosphogluconolactonase	**0.29**	*
64	**F6HGH4**	8	120.0	6-phosphogluconate dehydrogenase, decarboxylating	**9.40**	**
239	**D7TJI9**	4	50.4	Pyruvate decarboxylase	**0.34**	**
77	**F6HUI7**	8	88.3	RmlD_sub_bind domain-containing protein	**3.34**	*
**Cell wall (21)**						
131	**A0A438KK24**	6	75.1	Caffeoyl-CoA O-methyltransferase	**0.36**	**
54	**F6GSZ7**	9	119.1	Omega-hydroxypalmitate O-feruloyl transferase ^a^	**0.63**	*
**Amino acid metabolism (4)**						
3	**F6HMN8**	18	266.7	5-methyltetrahydropteroyltriglutamate--homocysteine S-methyltransferase	**2.90**	*
73	**A0A438GBL8**	8	106.4	Acetohydroxy-acid reductoisomerase	**6.64**	*
30	**F6I5Y5**	12	159.3	D-3-phosphoglycerate dehydrogenase	**1.66**	*
140	**F6H0X2**	6	67.6	Phospho-2-dehydro-3-deoxyheptonate aldolase	**2.39**	**
**Lipid metabolism (5)**						
51	**F6I1D6**	9	142.9	Non-specific phospholipase C3 ^a^	**0.52**	**
462	**A0A438FDG5**	2	20.2	Enoyl-CoA delta isomerase 3	**1.63**	*
**Secondary metabolism (9)**						
173	**F6HHQ7**	5	65.2	Putative acetyl-CoA acetyltransferase, cytosolic 2 ^a^	**0.39**	*
314	**A0A024FS61**	3	39.3	Polyphenol_oxidase	**4.73**	*
415	**A0A438ESC9**	2	25.1	3-isopropylmalate dehydratase small subunit 3	**0.09**	*
86	**F6I076**	7	107.7	CN hydrolase domain-containing protein	**0.05**	**
**Redox homeostasis (10)**						
185	**G1JT87**	5	59.7	Glutaredoxin-dependent peroxiredoxin	**0.41**	*
269	**D7T6T0**	4	39.8	Glutaredoxin-dependent peroxiredoxin	**4.16**	*
330	**A9UFY2**	3	36.1	Thioredoxin h-type	**0.45**	*
**Enzymes/Coenzyme metabolism (50, 7)**						
136	**D7TKJ3**	6	71.1	Ferredoxin-NADP reductase, chloroplastic	**new**	**
148	**D7SNB1**	6	58.9	Salutaridine reductase ^a^	**0.41**	*
11	**K9N4H5**	14	213.1	Mitochondrial aldehyde dehydrogenase 2B8	**0.65**	*
119	**A5BHH9**	6	80.8	NADH-cytochrome b5 reductase	**0.29**	*
41	**F6H5H5**	10	156.0	Trans-resveratrol di-O-methyltransferase ^a^	**1.63**	**
458	**A0A438CVH9**	2	20.5	UDP-glycosyltransferase 74F2	**7.77**	*
105	**A0A438KGT6**	6	98.7	Glucan endo-1,3-beta-D-glucosidase	**0.27**	**
151	**A0A438ITG1**	5	93.1	Putative cysteine protease RD21B	**0.36**	**
81	**A0A438EKJ2**	7	116.9	Phosphopyruvate hydratase (synonym: *Enolase*)	**2.28**	**
154	**A0A438D2Y0**	5	83.1	Phosphoglycerate mutase	**2.00**	**
38	**F6GTM7**	11	160.1	Adenosylhomocysteinase	**4.96**	*
**DNA/RNA/Cell cycle (6, 12, 13, 16)**						
280	**A5B6U5**	4	34.5	Proliferating cell nuclear antigen	**0.05**	*
378	**F6GSZ1**	2	31.5	RRM domain-containing protein	**0.61**	*
**Protein (17,18,19,23)**						
300	**A5AXI6**	3	43.0	60S acidic ribosomal protein P0	**24.11**	**
126	**A5BUU4**	6	78.2	40S ribosomal protein SA	**4.43**	**
217	**A5C4J2**	4	58.0	40S ribosomal protein S19-3 ^a^	**2.38**	*
443	**A5AJ83**	2	22.4	Ribosomal_S10 domain-containing protein	**4.27**	*
135	**A0A438KA42**	6	72.8	Guanine nucleotide-binding protein subunit beta-like protein	**3.44**	**
375	**F6HLE8**	2	33.4	Ribosomal_S7 domain-containing protein	**3.47**	*
171	**A0A438C2W6**	5	65.7	Aspartate-tRNA ligase	**2.44**	*
37	**F6HXZ5**	11	164.6	Eukaryotic initiation factor 4A-2 ^a^	**2.50**	**
407	**F6GTY8**	2	26.2	Tr-type G domain-containing protein	**10.31**	**
149	**A0A438CSH7**	6	57.0	Elongation factor 1-gamma	**new**	*
128	**F6H4T7**	6	77.2	Tr-type G domain-containing protein	**20.06**	**
346	**A0A438JTD3**	3	33.5	Dolichyl-diphosphooligosaccharide-protein glycosyltransferase 48 kDa subunit	**new**	*
44	**D7TBD9**	10	143.7	Alpha-MPP	**0.39**	*
99	**A5ANH8**	7	82.5	Probable mitochondrial-processing peptidase subunit beta, mitochondrial ^a^	**3.78**	*
176	**A0A438DUK9**	5	63.2	Protein disulfide-isomerase	**13.28**	**
335	**A0A438K994**	3	35.3	Citrulline-aspartate ligase	**7.32**	**
47	**D7SIX7**	10	128.0	Serine/threonine-protein phosphatase 2A 65 kDa regulatory subunit A beta isoform ^a^	**0.56**	*
108	**A0A438G7L8**	6	97.4	Glutathione S-transferase U10	**2.49**	*
337	**F6I510**	3	34.9	Putative glutathione S-transferase parC ^a^	**2.94**	*
448	**F6GT86**	2	21.5	Glutathione S-transferase ^a^	**4.05**	*
232	**A0A438KHW4**	4	53.1	Glutathione transferase	**1.65**	*
452	**F6HYG1**	2	21.4	Heat shock 70 kDa protein 15-like ^a^	**new**	*
1	**F6HNX5**	20	361.7	Putative heat shock cognate protein 2 ^a^	**2.04**	**
370	**A0A438K358**	2	38.1	Hsp70-Hsp90 organizing protein 1	**new**	**
317	**A0A438D490**	3	38.2	Heat shock cognate protein 80	**new**	*
107	**D7SLM9**	6	98.0	RuBisCO large subunit-binding protein subunit beta, chloroplastic ^a^	**5.54**	**
145	**F6GUM1**	6	65.0	E1 ubiquitin-activating enzyme	**6.77**	**
379	**A0A438J7X4**	2	31.5	Ubiquitin-conjugating enzyme E2-17 kDa	**1.48**	*
147	**A0A438KGZ1**	6	62.4	Proteasome subunit beta	**0.52**	*
420	**D7SKV3**	2	24.4	Proteasome subunit beta	**6.25**	*
388	**A0A438EWK5**	2	29.3	26S proteasome regulatory subunit 7	**8.65**	*
322	**F6HT17**	3	36.9	PCI domain-containing protein	**3.30**	*
93	**A0A438JN39**	7	93.2	Serine carboxypeptidase-like 7	**0.48**	*
115	**D7T3Q1**	6	85.9	Glucose acyltransferase 1 ^a^	**0.48**	**
195	**A5C1I0**	5	57.1	Carboxypeptidase	**0.71**	*
26	**F6H7H1**	12	172.6	Aspartic proteinase A1 ^a^	**0.56**	*
396	**A0A438K8Z1**	2	28.0	Aminopeptidase	**new**	**
400	**A0A438EKP3**	2	27.0	Ankyrin repeat domain-containing protein 2A	**0.21**	*
228	**A0A438JPS3**	4	53.8	GTP-binding nuclear protein Ran1B	**12.55**	**
**Cytoskeleton organization (20)**						
4	**A5ATG8**	17	307.8	Tubulin beta chain	**0.78**	*
347	**A0A438F6R2**	3	33.3	T-complex protein 1 subunit gamma	**4.31**	**
**Vesicle trafficking (22)**						
144	**D7T9L8**	6	65.0	Coatomer subunit delta	**6.58**	**
**Solute transport/Nutrient uptake (24, 25)**						
306	**F6I0Z8**	3	40.4	Plasma membrane 22 aquaporin	**2.81**	**
203	**Q9FS46**	4	70.6	Putative aquaporin	**0.65**	**
216	**A5AQ65**	4	58.1	Mitochondrial outer membrane protein porin 2 ^a^	**0.38**	**
325	**A0A438CTH2**	3	36.5	Mitochondrial outer membrane protein porin of 34 kDa	**0.43**	**
246	**A0A438FMR0**	4	46.7	Ferredoxin--nitrite reductase, chloroplastic	**new**	**
29	**A5AP38**	12	162.2	Glutamine synthetase (*cytosolic* ^a^)	**0.65**	*
321	**A0A438E3X6**	3	37.1	Ferritin	**10.77**	**
**Phytohormone action/External stimuli response (11, 26)**						
224	**F6H6V6**	4	54.5	Senescence-associated carboxylesterase 101 ^a^	**4.18**	**
**Not assigned-annotated (35.1)**						
46	**A0A438KRJ6**	10	138.5	Annexin (*D2* ^a^)	**1.44**	**
403	**A0A438JYU9**	2	26.9	Dipeptide epimerase	**8.06**	**
76	**D7SJF5**	8	95.2	Cystathionine beta-synthase family protein ^a^	**5.70**	**
158	**A5BM68**	5	73.7	TCTP domain-containing protein	**1.81**	*
48	**A0A438J6W5**	10	124.3	Glutelin type-A 2	**0.11**	**
230	**A0A438KKU7**	4	53.6	Stem-specific protein TSJT1	**0.26**	**
89	**A0A438JUJ6**	7	104.2	MLP-like protein 34	**1.49**	**
165	**A0A438JUL6**	5	68.5	MLP-like protein 43	**3.25**	*
42	**F6GTA6**	10	147.7	PHB domain-containing protein	**0.54**	**
91	**D7TNE5**	7	97.3	PHB domain-containing protein	**0.51**	*
106	**A0A438J2L0**	6	98.2	Chalcone-flavonone isomerase family protein (synonim: *Chalcone isomerase*)	**0.61**	*
110	**A0A438BSC8**	6	92.9	NAD(P)H dehydrogenase (quinone)	**0.40**	*
215	**D7T2N7**	4	59.9	Late embryogenesis abundant protein Lea14-A, putative ^a^	**0.58**	*
265	**D7T3J3**	4	41.3	Proline iminopeptidase	**0.26**	**
289	**F6H6H8**	3	48.8	Glyco_hydro_18 domain-containing protein	**d.**	*
387	**A5BM29**	2	29.4	NTF2 domain-containing protein	**0.11**	**
104	**D7T7N4**	6	102.7	RRM domain-containing protein	**0.28**	*
112	**A0A438GQU3**	6	91.0	Kunitz trypsin inhibitor 2	**0.02**	**
199	**A0A438IQU7**	5	47.6	Major allergen Pru ar 1	**d.**	*
180	**A0A438C6P2**	5	62.0	Plastid-lipid-associated protein, chloroplastic	**0.14**	*
395	**A5AJB3**	2	28.1	Chitin-binding type-1 domain-containing protein	**d.**	*
208	**A0A438JVD2**	4	64.1	Peroxidase	**0.42**	**
345	**F6HIK4**	3	33.9	Peroxidase	**4.52**	*
**Not assigned-not annotated (35.2)**						
72	**A5C8L8**	8	106.9	Pyr_redox_2 domain-containing protein	**5.10**	**
95	**D7TA35**	7	88.6	Usp domain-containing protein	**4.39**	*
273	**D7U4I8**	4	38.5	Usp domain-containing protein	**d.**	**
142	**A0A438JK35**	6	66.9	Bifunctional epoxide hydrolase 2	**0.01**	*
167	**A5AEX6**	5	67.0	DLH domain-containing protein	**0.75**	*

## Data Availability

The data presented in this study are available on request from the corresponding author. The mass spectrometry proteomics data are available via ProteomeXchange with identifier PXD025212.

## Supplementary Materials

The following are available online at https://www.mdpi.com/article/10.3390/plants10040792/s1, Table S1: Protein quantification by nLC–nESI–MS/MS in M4 root proteome, Table S2: Functional classification of the identified proteins in M4 rootstock genotype.
